# Two carbapenem-resistant ST1:ST231:KL1:OCL1 *Acinetobacter baumannii* strains recovered in Tehran, Iran, carry AbaR31 in the chromosome and AbaR4 and Tn*aphA6* in a RepAci6 plasmid

**DOI:** 10.1093/jacamr/dlab112

**Published:** 2021-08-07

**Authors:** Masoumeh Douraghi, Parisa Aris, Joyce To, Garry S A Myers, Mohammad Hamidian

**Affiliations:** 1 Division of Microbiology, Department of Pathobiology, School of Public Health, Tehran University of Medical Sciences, Tehran, Iran; 2 The iThree institute, University of Technology Sydney, Ultimo, NSW, Australia

## Abstract

**Objectives:**

To analyse the context of genes conferring antibiotic resistance in two carbapenem-resistant *Acinetobacter baumannii* isolates recovered in Tehran, Iran.

**Methods:**

The antibiotic resistance phenotype for 28 antibiotics was determined using disc diffusion. The whole genome sequences of ABH008 and ABS200 were determined using the Illumina HiSeq X Ten platform. Resistance genes were identified using ResFinder and multilocus sequence types were determined using the Oxford and Institut Pasteur schemes.

**Results:**

Isolates ABH008 and ABS200, recovered in 2012 and 2013, respectively, in two different Tehran hospitals, belong to the common global clone 1 lineage, ST1_IP_ and ST231_OX_. They are resistant to sulfamethoxazole, tetracycline, gentamicin, amikacin, third-generation cephalosporins and carbapenems. Despite being isolated in different hospitals, phylogenetic analysis indicated they are closely related. Consistent with this, both isolates carry *catA1, sul1, aacC1* and *aadA1* in a novel variant of the AbaR3-type resistance island, named AbaR31. Both isolates are resistant to amikacin and carbapenems owing to *aphA6* and *oxa23*, respectively. The *oxa23* gene is located in the AbaR4 resistance island, and *aphA6* in Tn*aphA6*, and both mobile elements are in an ∼90 kbp plasmid encoding the putative RepAci6 replication initiation protein. Resistance to third-generation cephalosporins is due to the acquisition by homologous recombination of a 5 kb DNA segment that contains ISAba1-*ampC* from a ST623 strain.

**Conclusions:**

The resistance gene complements of ABH008 and ABS200 were found in AbaR31 and a plasmid that encodes RepAci6. The close genetic relationship of ABH008 and ABS200, despite each being recovered from different hospitals, indicates transmission between the two hospitals.

## Introduction

Resistance to carbapenems in *Acinetobacter baumannii*, the number one WHO priority pathogen, is widespread, posing a global threat as carbapenems are the frontline option for treating infections caused by multi-antibiotic-resistant strains of this opportunistic pathogen.^1^^,^^2^ In *A. baumannii* carbapenem resistance is most often caused by acquired class D carbapenem oxacillinases, typically OXA-23, OXA-24 (OXA-40) and OXA-58.^2^^,^^3^

Two clonal complexes of *A. baumannii* are distributed globally: global clone 1 (GC1) and global clone 2 (GC2). Within each is a myriad of lineages and sub-lineages. The genetic variation among these lineages informs understanding of how resistance evolves and enables epidemiological tracking of antibiotic-resistant strains. For *A. baumannii*, the majority (70%) of the whole genome sequence data feeding variant analysis is geographically skewed.^2^ It is sourced from isolates obtained from only four countries, all outside the Middle East region^2^ while the source of many resistant lineages of *A. baumannii*, however, is believed to be the Middle East region.^4–7^ This discrepancy makes it difficult to draw an accurate global picture of *A. baumannii*. We recently reported analysis of whole genome sequence data from an outbreak in an Iranian hospital that supports the view that lineage 2 GC1 strains may have an origin in the region.^5^

Here, we report analysis of whole genome sequence data for two antibiotic-resistant *A. baumannii* strains isolated from two different hospitals in Iran. Both belong to lineage 1 of GC1 indicating that strains belonging to lineage 1 are also present in the country.

## Methods

### Antibiotic resistance phenotype and PCR mapping

Antibiotic resistance profiles of ABS200 and ABH008 were determined using the standard Kirby-Bauer disc diffusion assay to 28 antibiotics as previously described.^5^ Resistance to colistin was determined by measuring the MICs using the standard micro-broth dilution method.^8^ Antibiotic discs tested were ampicillin, streptomycin, spectinomycin, sulfamethoxazole, tetracycline, trimethoprim, kanamycin, neomycin, cefotaxime, ceftazidime, gentamicin, ciprofloxacin, amikacin, nalidixic acid, tobramycin, netilmicin, imipenem, meropenem, ticarcillin/clavulanic acid, rifampicin, ampicillin/sulbactam, cefepime, doripenem, piperacillin/tazobactam, ceftriaxone, minocycline, doxycycline and levofloxacin. Strains were classified as resistant or susceptible according to the CLSI guidelines for *Acinetobacter* spp.^9^ and Calibrated Dichotomous Sensitivity (CDS) disc diffusion assay (http://web.med.unsw.edu.au/cdstest) where an *Acinetobacter* spp. CLSI breakpoint was not available (e.g. for netilmicin, streptomycin, spectinomycin, sulfamethoxazole, nalidixic acid and rifampicin).

AbaR resistance islands were mapped using the PCR mapping strategy previously developed including the novel AbaR31 junction, which was mapped using the RH513: 5′-GTACTGTTGTAATTCATTAAGCAT-3′ and intI1-RV: 5′-GGGCATGGTGGCTGAAGGACC-3′ primers generating a 3 kb product.^10^^,^^11^

AbaR4 was also mapped using previously designed primers^12^ and located in a RepAci6 plasmid using two mapping PCRs (i) MH5 (5′-CTAAAAGGCGTTTGGGCATA-3′) and RH909 (5′-GCGATTCAAAATATCGGTCAA-3′)^13^ (generating a 3105 bp product) and (ii) MH7 (5′-TGCTGAACCGTACAACCAGA-3′) and MH6 (5′-CAGGATGAGCTGGATCAACA-3′) (generating a 3645 bp product) developed here. PCR conditions were the same as those described elsewhere.

### Whole genome sequencing, assembly and phylogenetic analysis

Genomic DNA isolated from ABH008 and ABS200 was sequenced using Illumina HiSeq X Ten. Paired-end reads of 150 bp were assembled using SPAdes (v. 3.14.1) yielding 75 and 77 contigs for ABH008 and ABS200, respectively, and with an average depth of ∼100-fold for each genome. Multi-locus Sequence Types in the Institut Pasteur (ST_IP_) and Oxford (ST_OX_) schemes as well as the *ampC* alleles were determined from the genome sequence data using the PubMLST database (http://pubmlst.org/abaumannii/).

Antibiotic resistance genes and contigs carrying them were identified using ResFinder (https://cge.cbs.dtu.dk/services/ResFinder/), and recovered using standalone BLAST (ftp://ftp.ncbi.nlm.nih.gov/blast/executables/blast+/LATEST/).

Genomes were annotated using the NCBI Prokaryotic Genome Annotation Pipeline (v.4.11).^14^ Pfam (http://pfam.xfam.org/) and ISFinder (https://www-is.biotoul.fr/) search tools were used to identify proteins and insertion sequences, respectively. Here, we used the ST_IP_#:ST_OX_#:KL#:OCL# formula that we recently developed^15^ to group/distinguish *A. baumannii* isolates.

**Figure 1.**
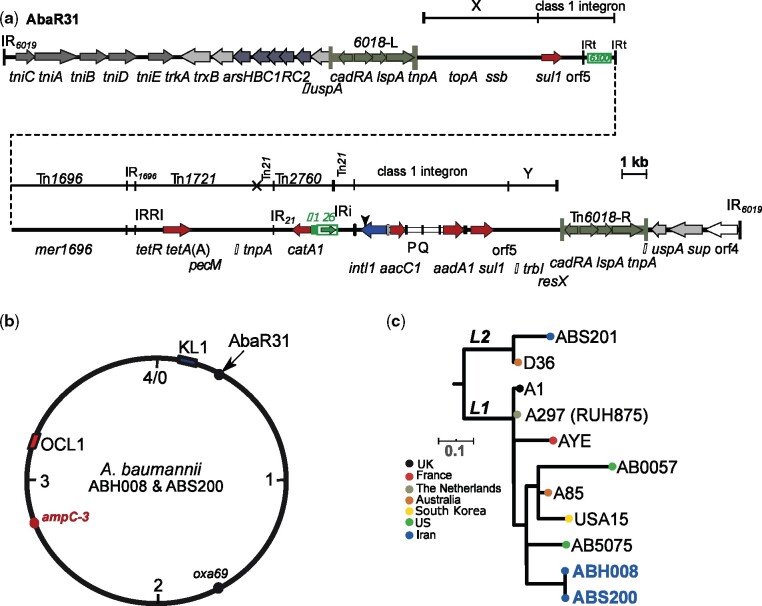
Structure of AbaR31 (a), circular map representing the chromosomes of ABH008 and ABS200 (b) and phylogenetic tree of representative GC1 strains (c). In (a), The thick central line shows the AbaR31 resistance island with horizontal arrows indicating the extent and orientation of genes. Antibiotic resistance genes are shown using red arrows. Two terminal vertical lines indicate the inverted repeats (IRs) of AbaR31. Tn*6018* copies are shown using dark green. The origin of each segment of the multiple antibiotic resistance region (MARR), flanked by Tn*6018* copies, is shown using a thin line above the central line. The vertical arrowhead above the *intI1* gene indicates the 108 bp deletion found in AbaR31. (b) Represents a schematic of the ABH008 and ABS200 chromosome with the position of AbaR31, *ampC*, K and OC loci marked. (c) Shows the phylogenetic relationship of ABH008 and ABS200 compared with known GC1 strains including GC1 reference strain A297 (RUH875).

Phylogenetic analysis was done by constructing a recombination-free tree by generating a whole genome alignment using snippy (v.4.6.0) (publicly available at https://github.com/tseemann/snippy) followed by removing regions potentially introduced by recombination using Gubbins^16^ (v2.1.025) as previously described in detail.^5^^,^^6^ A set of nine GC1 strains known to belong to lineage 1 and 2, including the A1 GC1 reference genome (GenBank accession numberCP010781), were also used as controls (Figure 1). Figures were drawn to scale using SnapGene^®^ (v. 5.2.4.) and Inkskape (v. 1.0).

### Nucleotide accession numbers

The genome sequence of ABH008 and ABS200 have been deposited in GenBank GenBank/EMBL/DDBJ database and are publicly available under the accession numbers JADPVA000000000 and JADPVB000000000.

## Results and discussion

### Resistance profiles, MLST and phylogenetic relationship

ABH008 and ABS200 are carbapenem-resistant isolates from two different hospitals in Tehran, Iran, isolated in 2012 and 2013, respectively. We determined their resistance phenotype and their relationship to global *A. baumannii* clones. ABH008 and ABS200 are resistant to carbapenems, third-generation cephalosporins, ticarcillin/clavulanic acid, tetracycline, streptomycin, spectinomycin, gentamicin, kanamycin, neomycin, amikacin sulphonamides, trimethoprim, nalidixic acid and ciprofloxacin, (Table S1, available as Supplementary data at *JAC-AMR* Online) while remaining susceptible to colistin (MIC <0.5 mg/L). Hence, they are considered extensively drug resistant (XDR).^17^ ABH008 and ABS200 genomes both contain the *catA1*, *tetA*(A)*, sul1, aacC1, aadA1, aphA6* and *oxa23* genes, accounting for the resistance phenotype observed. ABH008 and ABS200 were found to be GC1 based on analysis of the allelic-specific PCR^18^ which determines clones and their draft genomes. Consistent with other GC1s, both strains encode the OXA-69 variant of the intrinsic *oxaAb* gene and belong to ST1_IP_(1-1-1-1-1-5-1) and ST231_OX_(10-12-4-11-4-98-5). Phylogenetic analysis showed ABH008 and ABS200 were tightly clustered together, both falling within lineage 1 (Figure 1). It is possible that the ABH008 and ABS200 branch might represent the lineage 1 strains circulating in Tehran’s hospitals; however, more genome sequences would be needed to confirm this.

### ABH008 and ABS200 carry AbaR31; a novel antibiotic resistance island derived from AbaR3

To determine the genetic context of the resistance genes of ABH008 and ABS200, we used a combination of draft genomes and PCR mapping strategies.^10–13^ The *catA1*, *tetA*(A)*, sul1, aacC1, aadA1* resistance genes were localized in a novel variant of AbaR0/3-type islands with a Tn*6019* transposon backbone located in the *comM* gene of ABH008 and ABS200. Our draft genome analysis indicated that this island derives from AbaR3 rather than AbaR0 as it contains a 108 bp sequence in the 5′-CS of the class 1 integron, which is characteristic of AbaR3 (e.g. in AB0057; GenBank accession numberCP001182) and its variants.^19^ We named this new AbaR3 variant AbaR31. It is predicted to be 51 113 kbp (Figure 1). AbaR31 has been generated via an IS*26-*mediated adjacent deletion of an 11 877 bp internal segment of the AbaR3 island that includes the *bla*_TEM_ and *aphA1b* antibiotic resistance genes. IS*26*-mediated deletions are known to be important in generating variants of AbaR0/3 islands.^19^ We confirmed the novel junction in AbaR31 generated by the IS*26*-mediated deletion by manual sequencing of the ∼3 kbp product of a PCR that links *intI1* with *catA1*. The presence of AbaR31 in both ABH008 and ABS200 indicates their close relationship as well as transmission between hospitals.

### AbaR4 and TnaphA6 carried on a RepAci6 plasmid

The two antibiotic resistance genes of ABH008 and ABS200, *aphA6* and *oxa23*, that are not carried on the AbaR31 island were found in different transposons on the same plasmid. Using published primers^13^ we previously showed that the *aphA6* amikacin resistance gene is located in Tn*aphA6* inABH008 and ABS200 in a context similar to that seen in RepAci6 plasmids.^20^ Here, analysis of draft genomes confirmed that the *aphA6* gene is in Tn*aphA6*, in precisely the location that this transposon is in pAb-G7-2 (GenBank accession numberKF669606).^13^ The plasmid pAb-G7-2 is a RepAci6 plasmid found in an Australian GC1.^13^ All remaining segments of pAb-G7-2 were recovered in multiple contigs, indicating that the RepAci6 plasmid in ABH008 and ABS200 is closely related to pAb-G7-2 (with 99.9% DNA identities in plasmid backbone). The *oxa23* gene is in Tn*2006* in AbaR4; this was confirmed by retrieving all of the contigs making up the complete AbaR4 from draft genomes. Using the genome sequence data, AbaR4 was predicted to be located on the same RepAci6 plasmid that contains Tn*aphA6.* This prediction was confirmed by developing a PCR mapping strategy, followed by manual sequencing of products, to link unique sequences of either end of AbaR4 (*tniB* on the left and *oxa23* on the right) to the backbone of RepAci6 plasmid (see Methods). This led to the localization of AbaR4 in a spot equivalent to the position 9601 of pAb-G7-2 (GenBank accession numberKF669606) interrupting an open reading frame that encodes a hypothetical protein (protein id AGY56207.1). Sequence analysis also confirmed that AbaR4 is flanked by 5 bp target site duplication (TSD) 5′-AAAAG-3′, a typical property of transposons that belong to this family.^12^ AbaR4 has already been detected in RepAci6 plasmids, e.g. pA85-3 (GenBank accession numberCP021787); however, in the genomes of ABH008 and ABS200 it is present in a unique location in the plasmid backbone, which indicates local acquisition is likely. RepAci6 plasmids are potentially conjugative,^13^^,^^21–23^ and have been associated with the *aphA6* (amikacin resistance) and *oxa23* (carbapenem resistance) genes singly,^6^^,^^24^ or together,^23^ which combined makes RepAci6 plasmids important vehicles to spread amikacin and carbapenem resistance genes. To date, RepAci6 plasmids have not been seen in other Gram-negative bacteria e.g. enterobacteria or *Klebsiella pneumoniae*.

Analysis of the draft genomes of ABH008 and ABS200 also showed both strains contain a copy of a cryptic plasmid encoding RepAci1 that was identical to pA1-1 (GenBank accession numberCP010782) found in *A. baumannii* A1 recovered in the UK in 1982.^25^ The plasmid pA1-1 and its variants encode the AbkA (pfam14384) and AbkB (pfam04365) toxin–antitoxin system, which helps plasmids to be stably maintained in the cells. This plasmid type is widespread in *A. baumannii* strains regardless of clonal type and geographical distribution.^6^^,^^24^

### Resistance to third-generation cephalosporins due to acquisition of a DNA segment containing ISAba1-ampC

**Figure 2.**
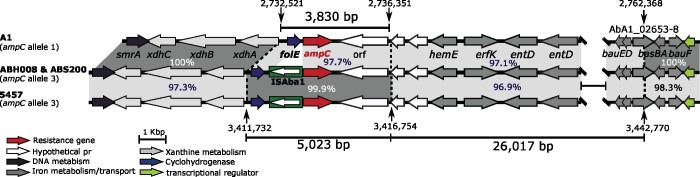
Schematic representation of the *ampC* region in ABH008 and ABS200 compared with A1 (GenBank accession numberCP010781) and 5457 (GenBank accession numberCP045541). The thick central line shows the chromosomes and horizontal arrows indicate the extent and orientation of genes. Shades of grey indicate regions with significant identity and numbers show their percentage DNA identity. Vertical arrows with numbers (above or below them) indicate chromosomal positions of A1 and 5457 as in CP010781 and CP045541, respectively.

The presence of ISAba1 upstream of the intrinsic *ampC* gene is known to be responsible for increasing the expression level of this gene, leading to resistance to third-generation cephalosporins.^26^ Both ABH008 and ABS200 include a copy of ISAba1 9 bp away from the start of *ampC*, explaining their resistance to ceftazidime and cefotaxime. However, analysis of the *ampC* sequences of ABH008 and ABS200 revealed that they do not include the standard ST1 *ampC* sequence (allele 1). Instead, they carry the allele 3 *ampC* sequence, typical in ST623 strains (e.g. strain 5457; GenBank accession numberCP045541). We have previously reported several cases of the acquisition of DNA segments containing an ISAba1-activated *ampC* gene by homologous recombination leading to third-generation cephalosporin resistance.^27^^,^^28^ Similarly, comparative analysis of the *ampC* region in ABH008 and ABS200 with the corresponding regions in A1 (GenBank accession numberCP010781 containing allele 1) and 5457 (GenBank accession numberCP045541 containing allele 3) also indicated that the ISAba1-*ampC* structure has been acquired from 5457 and incorporated into their chromosome as part of an ∼5 kbp DNA segment (Figure 2). A ∼26 kbp DNA patch was also found immediately adjacent to the 5 kbp fragment that contains ISAba1-*ampC* (Figure 2). This segment differed from the corresponding regions in A1 and 5457 by 97.1% and 96.9%, respectively (Figure 2). The source for this 26 kbp chromosomal segment could not be found. However, interestingly, an identical 5022 bp *ampC-3* patch, along with its adjacent ∼26 kbp segment, was also found in the *A. baumannii* strain DA33382 (GenBank accession numberCP030106), indicating it is closely related to ABH008 and ABS200. DA33382 is an ST1:ST1567:KL40:OCL2 GC1 strain recovered from a tracheal secretion sample in Germany with an unknown isolation date. The fact that DA33382 has a different KL and OCL, compared with ABH008 and ABS200, indicates that it has diverged by switching its outer core and capsular surface polysaccharides. We previously proposed that the Middle East region might be the source for many of the globally distributed resistance lineages of GC1s.^5^^,^^6^ The relationship of DA33382, ABH008 and ABS200 provides further evidence of this, but more genomes from the region will be needed to confirm the proposition.

### Conclusions

The ST1:ST231:KL1:OCL1 strains ABH008 and ABS200 belong to lineage 1 within global clone 1. Both strains are extensively antibiotic resistant due to the presence of AbaR31 and a RepAci6 plasmid that carries AbaR4 and Tn*aphA6*. ABH008 and ABS200 show that strains belonging to lineage 1 are circulating in Tehran hospitals and that they are closely related to a strain from Germany.

## Supplementary Material

dlab112_Supplementary_Data
