# Supplementary material for: Two carbapenem-resistant ST1:ST231:KL1:OCL1 Acinetobacter baumannii strains recovered in Tehran, Iran, carry AbaR31 in the chromosome and AbaR4 and TnaphA6 in a RepAci6 plasmid
Source: JAC Antimicrob Resist. 2021 Aug 7;3(3):dlab112. doi: 10.1093/jacamr/dlab112 (PMC8346695; doi:10.1093/jacamr/dlab112)
Supplement: dlab112_Supplementary_Data [file dlab112_supplementary_data.docx]

**Table S1.** Antibiotic susceptibility profile of the isolates tested.^a^

| **Isolate** | **Ap** | **Sm** | **Sp** | **Su** | **Tc** | **Tp** | **Km** | **Nm** | **CTX** | **CAZ** | **Gm** | **Cip** | **AK** | **Nx** | **Tm** | **Ne** | **Ipm** | **Mem** | **TIM** | **Rif** | **SAM** | **FEP** | **DOR** | **TZP** | **CRO** | **MIN** | **DOX** | **LVX** |
| --- | --- | --- | --- | --- | --- | --- | --- | --- | --- | --- | --- | --- | --- | --- | --- | --- | --- | --- | --- | --- | --- | --- | --- | --- | --- | --- | --- | --- |
| ABH008 | 7 ^b^ | 8 | 7 | 7 | 7 | 7 | 7 | 7 | 7 | 7 | 7 | 7 | 7 | 7 | 19 | 13 | 7 | 7 | 7 | 10 | 13 | 7 | 7 | 7 | 7 | 14 | 9 | 8 |
| ABS200 | 7 | 7 | 7 | 7 | 7 | 7 | 7 | 8 | 7 | 7 | 7 | 7 | 9 | 7 | 20 | 13 | 11 | 7 | 7 | 12 | 14 | 11 | 7 | 9 | 7 | 12 | 8 | 10 |

^a^ Abbreviations: Ap: Ampicillin, Sm: Streptomycin, Sp: Spectinomycin, Su: Sulfamethoxazole, Tc: Tetracycline, Tp: Trimethoprim, Km: Kanamycin, Nm: Neomycin, CTX: Cefotaxime, CAZ: Ceftazidime, Gm: Gentamicin, Cip: Ciprofloxacin, AK: Amikacin, Nx: Nalidixic Acid, Tm: Tobramycin, Ne: Netilmicin, Ipm: Imipenem, Mem: Meropenem, TIM: Timentin (Ticarcillin/clavulanic acid), Rif: Rifampicin, SAM: Ampicillin/sulbactam, FEP: Cefepime, DOR: Doripenem, TZP: Piperacillin/Tazobactam, CRO: Ceftriaxone, MIN: Minocycline, DOX: Doxycycline, LVX: Levofloxacin.

^b^ numbers indicate diameters (in millimeter) of growth inhibition zones.
